# Current Evidence on Computer-Aided Diagnosis of Celiac Disease: Systematic Review

**DOI:** 10.3389/fphar.2020.00341

**Published:** 2020-04-16

**Authors:** Adriana Molder, Daniel Vasile Balaban, Mariana Jinga, Cristian-Constantin Molder

**Affiliations:** ^1^Carol Davila University of Medicine and Pharmacy, Bucharest, Romania; ^2^Center of Excellence in Robotics and Autonomous Systems, Military Technical Academy Ferdinand I, Bucharest, Romania; ^3^Gastroenterology Department, Dr. Carol Davila Central Military Emergency University Hospital, Bucharest, Romania

**Keywords:** celiac disease, computer aided diagnosis, artificial intelligence, endoscopy, feature extraction

## Abstract

Celiac disease (CD) is a chronic autoimmune disease that occurs in genetically predisposed individuals in whom the ingestion of gluten leads to damage of the small bowel. It is estimated to affect 1 in 100 people worldwide, but is severely underdiagnosed. Currently available guidelines require CD-specific serology and atrophic histology in duodenal biopsy samples for the diagnosis of adult CD. In pediatric CD, but in recent years in adults also, nonbioptic diagnostic strategies have become increasingly popular. In this setting, in order to increase the diagnostic rate of this pathology, endoscopy itself has been thought of as a case finding strategy by use of digital image processing techniques. Research focused on computer aided decision support used as database video capsule, endoscopy and even biopsy duodenal images. Early automated methods for diagnosis of celiac disease used feature extraction methods like spatial domain features, transform domain features, scale-invariant features and spatio-temporal features. Recent artificial intelligence (AI) techniques using deep learning (DL) methods such as convolutional neural network (CNN), support vector machines (SVM) or Bayesian inference have emerged as a breakthrough computer technology which can be used for computer aided diagnosis of celiac disease. In the current review we summarize methods used in clinical studies for classification of CD from feature extraction methods to AI techniques.

## Introduction

Celiac disease (CD) is a systemic autoimmune disease driven by gluten ingestion in genetically susceptible individuals. At some point during their lifetime, some of the DQ2/DQ8 positive individuals become gluten intolerant and develop an autoimmune reaction in response to dietary gluten, leading to small bowel injury consisting in villous atrophy (VA) and crypt hyperplasia. Although it is one of the most common chronic digestive disorders, with prevalence rate of 1% worldwide (Ludvigsson et al., 2016), CD is severely underdiagnosed. This is due to the frequently mislabeling patients with irritable bowel syndrome, lack of awareness among medical professionals about the extra-intestinal presentations of the disease (Jinga et al., 2018) and missed opportunities to screen for CD such as first-grade relatives, high-risk groups and not least scoping the upper gastrointestinal tract for unrelated reasons. Un-diagnosed CD bears the risk of several complications (nutritional, fertility-related and even malignancy) and reduced quality of life (Fuchs et al., 2018). Although the diagnosis of adult CD is very clear cut (CD-specific serology and sampling of duodenal mucosa by upper gastrointestinal endoscopy) and access to diagnostic tools has improved considerably, CD remains heavily underdiagnosed, with only 15%–20% of patients being detected through current strategies.

In the setting of open-access endoscopy and increasing number of examinations worldwide (including on-demand procedures), some have considered using endoscopy as an opportunity for detection of unsuspected CD, by careful analysis of the small bowel mucosa and recognition of subtle markers of VA. In fact, a study has shown that up to 5% of CD patients have undergone a previous endoscopy examination in the years before the diagnosis, and this could be considered a missed opportunity to diagnose it earlier (Lebwohl et al., 2012). Thus, endoscopy can be viewed not only as a diagnostic tool to confirm the disease by tissue sampling, but also as a case-finding tool for CD. Some have even proposed random duodenal biopsies during all upper endoscopy examinations, but this has proven a low diagnostic yield for CD with a high burden for endoscopists and pathologists and lack of cost-effectiveness (Herrod and Lund, 2018). Thus, the interest has been changed over to a better selection of patients in whom biopsy sampling should be carried out and the way to do it is by detection of markers of VA during endoscopy. However, recognition of changes in the duodenal mucosa can be challenging, especially in the setting of patchy or mild disease (Balaban et al., 2015); in order to overcome the subjectiveness in detecting these endoscopic markers of VA and to better delineate the subtle mucosal changes seen in early CD, some have proposed the use of computer-based processing of endoscopic images for the detection of VA, which could trigger the examiner to perform biopsies for the diagnostic protocol of CD.

Endoscopy with biopsy is currently considered the gold standard for the diagnosis of adult CD. Computer-assisted systems for the diagnosis of CD could improve the whole diagnostic work-up, by saving costs, time and manpower and at the same time increase the safety of the procedure by avoiding biopsy sampling and prolonged sedation associated with the multiple biopsy protocol. Not least, this nonbiopsy protocol could translate into a longer life for the endoscope, by avoiding warn of the working channel of the scope. Also, the histological staging of biopsies is subject to a significant degree of intra- and interobserver variability (Weile et al., 2000; Corazza et al., 2007; Mubarak et al., 2011; Arguelles-Grande et al., 2012). A further limitation of the endoscopy biopsies for the diagnosis of CD is due to the possibly patchy distribution of CD (Bonamico et al., 2004; Hopper et al., 2007), areas affected by CD can be in the midst of normal mucosa. So, given the case that the biopsies would be targeted from areas of healthy mucosa, CD could be missed due to a sampling error. Therefore, observer independent diagnostic methods such as computer-assisted diagnosis systems are very useful to improve the accuracy of diagnosis.

From the first research focused on computer-assisted system in the context of automated diagnosis of CD which has started in 2008 (Vécsei et al., 2008), over 50 publications on the topic that are using spatial domain, transform domain, scale-invariant and and spatio-temporal features have appeared (Hegenbart et al., 2015) but artificial intelligence (AI), machine learning (ML) and deep learning (DL) have emerged as a breakthrough computer technology in this world of big data and computational power based on graphics processing units. In the field of medical images, the accumulation of enormous digital images and medical records drove a need for the utilization of AI to efficiently deal with these data, which also become fundamental resources for the machine to learn by itself. ML and AI techniques have played an important role in the medical field, supporting such activities as medical image processing, computer-aided diagnosis, image interpretation, image fusion, image registration, image segmentation, image-guided therapy, and image retrieval and analysis (Razzak et al., 2018; Yang and Bang, 2019). In this work, we try to give a comprehensive overview of the research focused on computer-assisted diagnosis of CD from classical features extraction to AI. Several image-processing techniques have been reported so far in the literature, with good diagnostic performance in discriminating CD patients from healthy controls. Applying these image-processing techniques could be used to select in real-time, during endoscopy, patients with high probability of CD, who warrant a full diagnostic work-up including small bowel biopsies. The purpose of our review is to summarize current evidence of computerized methods in detecting CD, according to their diagnostic accuracy.

## Methods

We conducted the present research according to the principles of the preferred reporting items for meta-analysis protocol (PRISMA) (Moher et al., 2015).

A systematic search of the literature was carried out in September 2019 in PubMed (Medline) database, using the following search criteria: *CD* (*Mesh*) and terms referring to computer-aided detection by image processing – *computer*, *digital*, *image processing*, *AI*, *DL*, *neural network*, *quantitative assessment* or *texture features*. There were no restrictions set on the search with regard to article type, text availability or publication date.

Publications revealed through this search were assessed for consistency with the topic, according to their title and abstract, by the two first authors. Conflicts resulted from independent data extraction according to inclusion and exclusion criteria were resolved by consensus.

Studies included in our systematic review were required to meet the following inclusion criteria: (i) full-text paper available in English, (ii) original papers describing image-processing techniques for computer-aided diagnosis of CD. We excluded case-reports, reviews and descriptive papers without validation of methods described on CD patients. We also excluded papers referring to digital processing of histology images in diagnosing CD. References from the retrieved articles were also checked for possible match with the review topic, in order to identify potentially relevant publications that could have been missed on the initial search.

For each study included in the systematic review we recorded the following data: first author, year of publication, type of endoscopic image used, study population (CD cases and controls), method tested and diagnostic performance (sensitivity, specificity, diagnostic accuracy).

## Results

**Figure 1:**
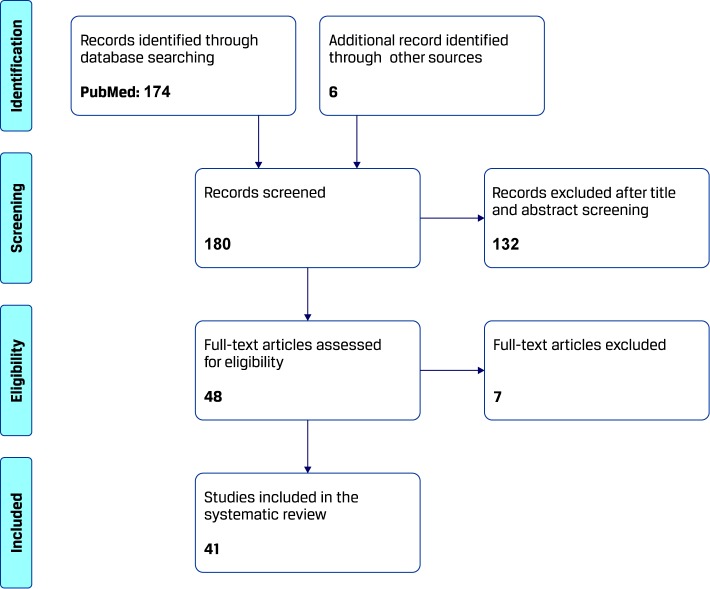
Search algorithm on computer-aided diagnosis of celiac disease (CD).

The process of study selection for this systematic review is summarized in **Figure 1**. The search yielded 174 results from 1970 onward, which were evaluated according to the above described methodology. Another six papers were found through other sources. A total of 139 papers were excluded because of irrelevance to the topic (confounding use of search words in the papers) or type of article (review/editorial) and the remaining 41 publications, consisting in original work describing image processing techniques for computer-aided diagnosis of CD, were analyzed for this review.

Among published papers, several techniques have been validated for the diagnosis of CD—first attempts were focused on features extraction methods used for classification such as spatial domain features, transform domain features, scale-invariant features and spatio-temporal features. More recent AI techniques DL methods such as convolutional neural network (CNN) have emerged as a breakthrough computer technology which can be used for computer aided diagnosis of CD. A statistic of common methods is presented, as well as an evaluation of their use in CD diagnosis.

### Feature Extraction Methods

**Table 1** summarizes the feature extraction methods used in clinical studies for the classification of the CD and the overall classification rates (OCR) [sensitivity (sens), specificity (spec) and accuracy (acc)]. Features can be classified into four main categories: spatio-domain features, transform domain features, scale-invariant features, and spatio-temporal features. Images used for feature extraction are obtained either by standard endoscopy or using video-capsule. While some of the studies reported the number of subjects analyzed (healthy and with CD), others have reported the number of full images and the number of subimages obtained as patches from the full images that were used for training and testing.

**Table 1:** Summary of features extraction methods used in clinical studies for classification of celiac disease (CD).

Reference	Published Year	Type of endoscopic images	Number of subjects (Database)	Method	OCR
**Spatial domain features**					
Gschwandtner et al. (2010)	2010	standard	Control: 153 image patches Celiac: 120 image patches	Edge- Shapes ELBP ELBP- Delaunay LTP	Edge-Shapes: 95.0% ELBP: 94.1% LBP-Delaunay: 61.6% LTP: 94.1%
Hegenbart et al. (2011b)	2011	standard	Control: 587 image patches Celiac: 412 image patches	LTP ELBP LBP/C	LTP: 98.9% ELBP: 98.9% LBP/C: 98.0%
Hegenbart et al. (2011c)	2011	standard	Control: 306 image patches from 131 patients Celiac: 306 image patches from 40 patients	LBP ELBP ELTP	LBP: sens 94.2%, spec 93.6%, acc 93.9% ELBP: sens 93.6%, spec 94.3%, acc 93.9% ELTP: sens 93.6%, spec 94.3%, acc 93.9%
Vécsei et al. (2011)	2011	standard	Control: 306 image patches from 131 patients Celiac: 306 image patches from 40 patients	LBP LTP LBP/C ELBP WT-LBP	LBP: sens 87.3%, spec 79.5%, acc 83.3% LTP: sens 94.0%, spec 75.5%, acc 84.7% LBP/C: sens 92.6%, spec 82.1%, acc 87.3% ELBP: sens 92.6%, spec 79.5%, acc 86.0% WT-LBP: sens 90.6%, spec 85.4%, acc 88.0%
Ciaccio et al. (2011)	2011	video-capsule	Control: 10 patients Celiac: 10 patients (200 frames/patients)	morphological skeletonisation	acc 64%
Hämmerle-Uhl et al. (2012)	2012	standard	Control: 304 image patches from 132 patients Celiac: 303 image patches from 54 patients	GLCM EOH	GLCM: sens 77%, spec 81%, acc 79% EOH: sens 73%, spec 72%, acc 72%
Hegenbart et al. (2012a)	2012	standard	Control: 86 images patches from 74 patients Celiac: 263 image patches from 74 patients	LBP ELBP	LBP: sens 68.5%, spec 90.7%, acc 79.2% ELBP: sens 79.4%, spec 86.1%, acc 82.6%
Hegenbart et al. (2012b)	2012	standard	Control: 306 images patches from 131 patients Celiac: 306 image patches from 40 patients	LBP LTP ELBP ELTP WT-LBP	LBP: sens 90.6%, spec 79.5%, acc 85.0% LTP: sens 83.2%, spec 75.5%, acc 79.3% ELBP: sens 94.0%, spec 74.2%, acc 84.0% ELTP: sens 92.0%, spec 73.2%, acc 83.0% WT-LBP: sens 92.6%, spec 85.4%, acc 89.0%
Gadermayr et al. (2013a)	2013	standard	Control: 163 image patches from 100 images from 59 patients Celiac: 124 image patches from 67 images from 23 patients	SCH ECM Haralick features SSD LBP LTP	SCH: 86.1% ECM: 86.1% Haralick features: 86.8% SSD: 90.2% LBP: 88.2% LTP: 86.8%
Gadermayr et al. (2013c)	2013	standard	Control: 163 image patches Celiac: 124 image patches	LBP LTP RLBP	LBP: 92.3% LTP: 92.3% RLBP: 90.2%
Gadermayr et al. (2013b)	2013	standard	Control: 306 image patches from 234 images from 131 patients Celiac: 306 image patches from 172 images from 40 patients	SCH	sens 85.3%, spec 89.9%, acc 87.8%
Gadermayr et al. (2014b)	2014	standard	Control: 306 image patches from 131 patients Celiac: 306 image patches from 40 patients	LBP LTP ELBP RLBP	LBP: approx. 79% LTP: approx. 90% ELBP: approx. 80% RLBP: approx. 78%
Kwitt et al. (2014)	2014	standard	Control: 592 images patches from 240 patients Celiac: 458 images from 80 patients	Multiscale LBP	acc 86%
Gadermayr et al. (2015)	2015	standard	Control: 306 image patches from 131 patients Celiac: 306 images from 40 patients	SH-LBP	acc 91%
Gadermayr et al. (2016a)	2016	standard	Control: 679 image patches from 215 patients (children) Celiac: 479 image patches from 75 patients (children)	MR-LBP	acc 92.8% hybrid system with expert increased acc to 98.9%
Gadermayr et al. (2016b)	2016	standard	Control: 840 image patches Celiac: 840 image patches	LBP LTP SCH	acc 93% (hybrid system)
Gadermayr et al. (2016)	2016	standard	Training: image patches (306 celiac, 306 control) Testing: 172 images from 72 patients	LBP ELBP SCH	acc 86%
**Transform domain features**							
Vécsei et al. (2008)	2008	standard	Control: 312 image patches Celiac: 79 image patches	WPC WT-BBC WT-LDB	WPC: 90.1% WT-BBC: 98.5% WT-LDB: 91.1%
Hegenbart et al. (2009)	2009	standard	Control: 612 image patches Celiac: 387 image patches	DT-CWT DT-CWT-Weibull WPC WT-Gabor WT-GMRF FFT-Evolved	DT-CWT: 91.2% DT-CWT-Weibull: 86.7% WPC: 86.0% WT-Gabor: 89.3% WT-GMRF: 89.8% FFT-Evolved: 93.2%
Vécsei et al. (2011)	2009	standard	Control: 312 image patches Celiac: 79 image patches	FFT- Evolved (multiple ring-shape filters)	sens 83%, spc 99%, acc 97%
Gschwandtner et al. (2010)	2010	standard	Control: 153 image patches Celiac: 120 image patches	CWT- Weibull WT-Gabor WT-BBC WT-GMRF FFT-Evolved	CWT-Weibull: 97.6% WT-Gabor: 95.5% WT-BBC: 90.8% WT-GMRF: 91.6% FFT-Evolved: 96.6%
Vécsei et al. (2011)	2011	standard	Control: 306 image patches from 131 patients Celiac: 306 images patches from 40 patients	DT-CWT DT-CWT-Weibull WT-Gabor WT-BBC WT-GMRF WT-LDB	DT-CWT: sens 81%, spec 83%, acc 82% DT-CWT-W: sens 77%, spec 87%, acc 82% WT-Gabor: sens 81%, spec 80%, acc 80% WT-BBC: sens 85%, spec 80%, acc 83% WT-GMRF: sens 85%, spec 75%, acc 80% WT-LDB: sens 87%, spec 79%, acc 83%
Liedlgruber et al. (2011)	2011	standard	Control: 125 image patches Celiac: 111 image patches	WT-DWT WT-BBC WT-LDB WT-GMRF FFT-Evolved	WT-DWT: 86.4% WT-BBC: 89.8% WT-LDB: 91.9% WT-GMRF: 91.5% FFT-Evolved: 97.0%
Gadermayr et al. (2013a)	2013	standard	Control: 163 image patches from 100 images from 59 patients Celiac: 124 image patches from 67 images from 23 patients	WPC WT-BBC WT-LDB WT-GMRF WT-GMRF-CNH	WPC: 72.8% WT-BBC: 77.0% WT-LDB: 79.8% WT-GMRF: 84.7% WT-GMRF-CNH: 88.5%
Kwitt et al. (2014)	2014	standard	Control: 592 image patches from 240 patients Celiac: 458 image patches from 80 patients	DT-CWT	acc 85%
Gadermayr et al. (2016b)	2016	standard	Control: 840 image patches Celiac: 840 image patches	DT-CWT	acc 93% (hybrid system)
Gadermayr et al. (2016)	2016	standard	Training: image patches (306 celiac, 360 control) Testing:172 images from 72 patients	Fourier Power Spectra Rings	acc 86%
Koh et al. (2019)	2019	video- capsule	Control: 13 patients Celiac: 13 patients	DWT	sens 89.8%, spec 82.3%, acc 85.9%
Vicnesh et al. (2019)	2019	video- capsule	Control: 702 image patches from 16 patients Celiac: 1027 image patches from 21 patients	DAISY descriptors	sens 94.4%, spec 83.2%, acc 89.8%
**Scale-invariant features**							
Uhl et al. (2011)	2011	standard	Control: 285 image patches Celiac: 284 image patches	SI Wavelet	acc 95.2%
Hegenbart et al. (2013)	2013	standard	Control: 306 image patches from 131 patients Celiac: 306 image patches from 40 patients	SI Wavelet SI fractal analysis SIFT Pulse-coupled NN Multiscale blob Affine invariant LTP	Fractal analysis 91.7% (best result)
Kwitt et al. (2014)	2014	standard	Control: 592 images from 240 patients Celiac: 458 images from 80 patients	SIFT	acc 86%
Gadermayr et al. (2016)	2016	standard	Training: image patches (306 celiac, 306 control) Testing: 172 images from 72 patients	MFS	acc 86%
Gadermayr et al. (2016a)	2016	standard	Control: 676 image patches from 215 patients (children) Celiac: 479 image patches from 75 patients (children)	MFS SIFT Fisher vectors	MFS: 96.8% SIFT Fisher vectors: 98.1% (hybrid system with expert increased acc to 98.9%)
Gadermayr et al. (2016b)	2016	standard	Control: 840 images patches Celiac: 840 image patches	MFS SIFT Fisher vectors	acc 93% (hybrid system)
**Spatio-temporal features**							
Ciaccio et al. (2010a)	2010	video-capsule	Control: 10 patients Celiac: 11 patients	Pixel brightness	Threshold class.: sens 80%, spec 96% Incremental class.: sens 88%, spec 80%
Ciaccio et al. (2010b)	2010	video-capsule	Control: 10 patients Celiac: 11 patients	Pixel brightness	sens 92.7%, spec 93.5%
Ciaccio et al. (2012b)	2012	video-capsule	Control: 10 patients Celiac: 11 patients	Dynamic estimation of wall motility (standard deviation)	sens 98.2%, spec 96.0%
Ciaccio et al. (2012a)	2012	video-capsule	Control: 11 patients Celiac: 12 patients	The tallest peak in the ensemble average power spectrum	71%
Ciaccio et al. (2013a)	2013	video-capsule	Control: 10 patients Celiac: 10 patients	Shape-from-shading	64%
Ciaccio et al. (2013b)	2013	video-capsule	Control: 7 patients Celiac: 9 patients	Pooling protocol	sens 83.9%, spec 92.9%, acc 88.1%
Ciaccio et al. (2014b)	2014	video-capsule	Control: 13 patients Celiac: 13 patients	Histogram mean level	sens 84.6%, spec 92.3%
Ciaccio et al. (2014a)	2014	video-capsule	Control: 7 patients Celiac: 9 patients	Texture subbands Motility estimation Shape-from-shading	sens 80%, spec 80%
Ciaccio et al. (2017a)	2017	video-capsule	Control: 8 patients Celiac: 8 patients	Shape-from-shading (elevation, standard deviation, brightness units)	sens 80%, spec 80%

The spatial domain features that were used for CD classification are the edge shapes, shape curvature histograms (SCH), gray-level cooccurence matrix (GLCM), edge orientation histogram (EOH), local binary patterns (LBP) and extended LBP (ELBP), local ternary patterns (LTP) and extended LTP (ELTP), LBP in wavelet subbands (WT-LBP), rotation-invariant LBP (RLBP), LBP combined with a contrast measure (LBP/C), soft histogram LBP (SH-LBP), and multiresolution LBP (MR-LBP).

Transform domain features used in studies were pyramidal Wavelet transform (WPC), best-basis centroids base (WT-BBC), local discriminant basis (WT-LDB), Wavelet-based Gaussian Markov random fields (WT-GMRF), WT-GMRF with custom neighborhoods (WT-GMRF-CNH), dual-tree complex Wavelet transform correlation signature (DT-CWT), DT-CWT-Weibull, Gabor Wavelet transform (GWT), best-basis decomposition of Wavelet transform (WT-BBC), Fourier power spectra rings, and DAISY descriptors.

Common scale-invariant features are scale invariant wavelet based features (SI-WTF), scale invariant methods based on fractal analysis (SI-FA), scale-invariant feature transform (SIFT), multiscale blob features (MBF), affine invariant LTP (AILTP), multifractal spectrum (MFS), and SIFT Fisher vectors.

Among spatio-temporal features one can mention the pixel brightness, dynamic estimate of wall motility (Bassotti et al., 1994; Tursi, 2004) (standard deviation), periodicity in brightness, histogram mean level, shape-from-shading, pooling protocol, and statistical and syntactical measurements. Ciaccio et al. proposed some quantitative measurement (statistical and syntactical measurement, motility estimation) in video capsule endoscopy images in order to detect and measure the presence of VA in CD patients (Ciaccio et al., 2016a; Ciaccio et al., 2016b). Also in (Ciaccio et al., 2017b), Ciaccio et al. described and discussed methods used for quantitative detection and analysis of VA in the small intestinal mucosa of CD patients using video capsule endoscopy images but these remain to be further validated in larger samples (Ciaccio et al., 2017b).

Best results obtained using spatial domain features were obtained by Hegenbart et al. (Hegenbart et al., 2011b) by extracting various LBP from standard endoscopic images. In a database consisting of 999 image patches (587 control and 412 CD), overall classification rates varied between 98.04% and 98.93%.

In terms of transform-domain features best results were obtained by Vécsei et al. (2009) using FFT-evolved multiple ring-shape filters applied on standard endoscopic images. Database consisted of 390 image patches (312 control and 79 CD). The algorithm proved 97% accuracy, 83% sensitivity and 99% specificity in diagnosing CD.

Best results for scale-invariant features were obtained by Gadermayr et al. (2016a) using multifractal spectrum features and SIFT Fisher vectors extracted from standard endoscopic images. Database consisted of 676 control image patches and 479 CD image patches from 290 patients (all children). Performance of the method was 98.1% for SIFT Fisher vectors and 96.8% for multifractal spectrum features. When human knowledge was incorporated, performance increased to 98.9%.

Best results for spatio-temporal features were obtained by Ciaccio et al. (2012b) using dynamic estimate of wall motility (standard deviation) computed on video-capsule endoscopic images. Database consisted of 200 frames per patient extracted from 10 control patients and 11 CD patients. Diagnostic performance was high, with 98.2% sensitivity and 96% specificity.

Gadermayr et al. in (2015) compared different endoscopic image configuration [white-light imaging (WLI) (Gasbarrini et al., 2003) and narrow-band imaging (NBI) (Emura et al., 2008; Valitutti et al., 2014)] to find which image data are most accurate in case of computer aided CD diagnosis but in (Gadermayr et al., 2016a; Gadermayr et al., 2016b) same authors et al. showed that an hybrid system which incorporated expert knowledge in automated CD diagnosis increased the accuracy with 6% (see **Table 1**).

Spatial domain features were based on the similarity of specific features also observed by a human analyst; they are robust and fast in terms of computation, making them suitable for real time classification. Transform domain features have the advantage of analysing endoscopic images on multiscale and multiorientation levels. Common tools are based on Wavelet transform, Fourier transform and Gabor transform. Scale-invariant features are suitable to analyse characteristics that are affected by scaling, but are more demanding in terms of computation than previous features. Spatio-temporal features have higher robustness to features that are not visible directly on the acquired images. In terms of computation, the more complex the feature extraction algorithm is, the more are suitable to an offline analysis. All extracted features presented herein are further used as inputs to various classifiers, such as: support vector machine (SVM), k-nearest neighbors (kNN), Bayes classifiers, and random forests. All these classifiers are standard pattern recognition techniques.

### AI Techniques

**Figure 2:**
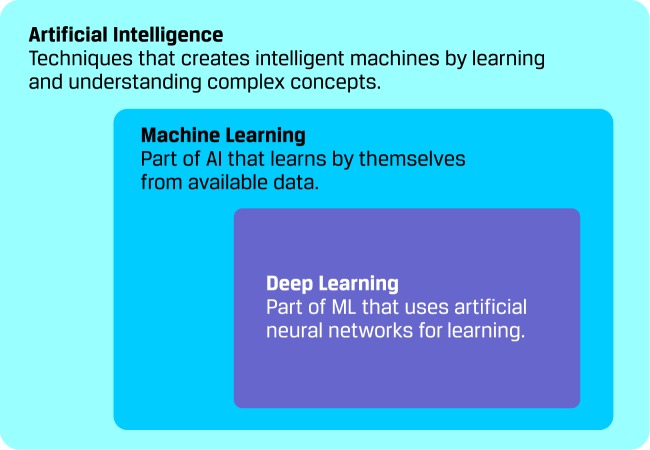
Machine learning as a branch of artificial intelligence.

The AI is the field of computer science that aims to create intelligent machines by learning and understanding complex concepts. As an AI branch, ML deals with intelligent machines that learns by themselves from available data. Moreover, DL refers to a family of ML methods that uses neural networks for learning (see **Figure 2**). These artificial neural networks (ANN) are biological-inspired computing systems that allows computers to learn.

In field of medical image processing there are some application that use AI. In Yang and Bang’s review (Yang and Bang, 2019) about applications of AI in gastroenterology, which summarizes clinical studies that are using AI in the upper and lower gastrointestinal field, there is a sole mention of CD. The review presented only Zhou’s study which has achieved a sensitivity and specificity of 100%. Seguí et al. in (Seguí et al., 2016) presented a generic feature descriptor for the classification of video capsule endoscopy images. In order to build the system they created a large database containing only color images, designed a CNN architecture and performed an exhaustive validation of the proposed method. They achieved very good results: 96% accuracy. Gadermayr et al. in (Gadermayr et al., 2018) investigated the capability of state-of-the-art neural network approaches for diagnosis of CD and proposed pipelines for fully-automated patient-wise diagnosis as well as for integrating expert knowledge into the automated decision process.

The availability of big data and computational power have led to the use of AI in medical applications on a large scale. **Table 2** summarizes AI techniques used in clinical studies for classification of CD. Common neural networks used in studies made for CD diagnosis are the AlexNet (Wimmer et al., 2016b; Yang and Bang, 2019), GoogLeNet (Zhou et al., 2017), VGGf net (Wimmer et al., 2016b; Gadermayr et al., 2017; Wimmer et al., 2017; Wimmer et al., 2018), and VGG16 net (Wimmer et al., 2016b; Wimmer et al., 2018). Only a single study used video-capsule images (Zhou et al., 2017), while all the others researches used standard endoscopic images. Comparing to the feature extraction, databases used in AI are based on a much larger number of patients [e.g., 353 patients in (Wimmer et al., 2016b)].

**Table 2:** Summary of AI techniques used in clinical studies for classification of celiac disease (CD).

References	Published year	Type of endoscopeic images	Number of subjects	Database	Type of AI	Outcomes
Tenório et al., (2011)	2011		120 control 96 celiac		Decision trees Bayesian classifiers ANN SVN kNN	acc: 84.2% sens: 92.9% spec: 79.2%
Wimmer et al., (2016b)	2016	standard	353 patients	986 control image patches 675 celiac image patches	CNN (AlexNet, VGG net) CNN SoftMax SVM	acc: 90.5%
Wimmer et al., (2016a)	2016	standard	353 patients	986 control image patches 675 celiac image patches	CNN with SVM and PCA	acc: 97.0%
Wimmer et al., (2017)	2017	standard	353 patients	986 control image patches 675 celiac image patches	CNN (VGGf net) CNN SoftMax SVM	acc: 91.5%
Gadermayr et al., (2017)	2017	standard	73 control 23 celiac	292 control images 92 celiac images Training and testing set: 72 patches/images	CNN (VGGf net) Adapted-CNN Non-adaptet-CNN Combined-CNN	acc: 90.3% sens: 92.9% spec: 87.6%
Zhou et al., (2017)	2017	video-capsule	10 control 12 celiac (1/2 for training, 1/2 for testing)	200 frames (512x512)x 4 regions/patients	CNN (GoogLeNet)	sens: 100% spec: 100%
Wimmer et al., (2018)	2018	standard	353 patients	986 control image patches 675 celiac image patches	CNN (AlexNet, VGGf net, VGG16 net) SVM	acc: 92.5%

Best results have been obtained on video-capsule images by Zhou et al. using GoogLeNet (Zhou et al., 2017). Although the database was reduced in terms of number of patients, 400 images were used for training that led to 100% specificity and 100% sensitivity. Best results on standard endoscopy images were obtained by Wiemer et al. using CNN with SVM and principal component analysis (PCA) (Wimmer et al., 2016a). They obtained a 97% good classification rate based on 1661 image patches (986 control and 675 CD).

## Discussion and Limitations

Even if the gold standard for the diagnosis of CD is considered to be the duodenal biopsy, advanced endoscopic techniques such as chromoendoscopy and water-immersion have been researched as enhanced tools to detect VA. The most notable techniques include the modified immersion technique (MIT) (Gasbarrini et al., 2003) under traditional white-light illumination (denoted as WL_MIT_), as well as MIT under narrow band imaging (Emura et al., 2008; Valitutti et al., 2014) (denoted as NBI_MIT_). These endoscopic techniques were specifically designed for improving the visual confirmation of CD during endoscopy. Other studies have proposed the use of video capsule images processing in detecting CD (Ciaccio et al., 2010a; Ciaccio et al., 2010b; Ciaccio et al., 2012a; Ciaccio et al., 2012b; Ciaccio et al., 2013b; Ciaccio et al., 2014b; Ciaccio et al., 2017a). Although it is considered as a noninvasive technique, its use is relatively low due to high cost and low resolution of image samples (de Bruaene et al., 2015).

One of the most important issues that are encountered in endoscopic image analysis is related to degradations such as noise, reflections, blurring and scaling due by weak illumination and downsized sensors. Some of the papers proposed some methods of improving these degradations (Hegenbart et al., 2011a; Hegenbart et al., 2011b; Gadermayr et al., 2014a).

Database construction is a critical subject in the AI-based classification of CD using endoscopic imagery. When large databases are not available, one can use data augmentation to artificially increase the number of samples of the database (Wimmer et al., 2017).

A major limitation of using automated-processing of duodenal images captured during endoscopy for diagnosing CD is the wide differential diagnosis of VA. Several other diseases other than CD can manifest as VA in the small bowel—giardiasis, Helicobacter pylori infection, Whipple’s disease, tropical sprue, collagenous sprue, eosinophilic gastroenteritis, common variable immunodeficiency, intestinal lymphoma, Crohn’s disease, HIV-enteropathy, or drug-induced enteropathy (Jinga et al., 2017; Schiepatti et al., 2019). Upon detection of VA by digitally processing of endoscopy images, CD-specific serology will discriminate CD from other causes of seronegative VA.

Another limit of using computerized automation methods for image processing is that of artifacts due to the presence of air bubbles, residues or secretions in the duodenum. This could represent an issue for selection of images frames which are processed for assessment of VA. Not least, selection of point of interest (POI) regions from the image captured during endoscopy is yet to be automatized; this could be considered a selection bias in current studies, as it was done manually during image processing. Also, a special concern is to be raised for cases with mild enteropathy, when changes in the duodenum are subtle, and those with patchy disease, when selection of the POI could be nonrepresentative.

Another limitation of the current review is the heterogeneity of studies with respect to cases analyzed, as some of the studies report the number of patients included, while others the number of images processed.

## Conclusion

In the last decades, there’s been a growing interest in image processing techniques for detection of VA. Computer-aided diagnosis of CD by processing images of the small bowel captured during endoscopy is feasible and warrants further development for integration into endoscopy consoles.

## Author Contributions

AM and DB discussed the research idea. C-CM and MJ proposed the research design. AM and DB performed the literature search and selection of articles. AM and DB drafted the first manuscript. MJ and C-CM critically reviewed the manuscript, acting as guarantors of the paper. All authors approved the final version of the manuscript.

## Funding

This paper was financially supported by “Carol Davila” University of Medicine and Pharmacy through Contract no. 23PFE/17.10.2018 funded by the Ministry of Research and Innovation within PNCDI III, Program 1 – Development of the National RD system, Subprogram 1.2 – Institutional Performance – RDI excellence funding projects.

## Conflict of Interest

The authors declare that the research was conducted in the absence of any commercial or financial relationships that could be construed as a potential conflict of interest.
